# Introduction

**DOI:** 10.1098/rstb.2008.0103

**Published:** 2008-11-27

**Authors:** John Collinge, Michael P. Alpers

**Affiliations:** 1 MRC Prion Unit and Department of Neurodegenerative Disease, UCL Institute of Neurology, The National Hospital for Neurology and Neurosurgery, Queen SquareLondon WC1N 3BG, UK; 2 Centre for International Health, ABCRCHealth Research Campus, Shenton Park, Curtin University, GPO Box U1987, Perth, WA 6845, Australia

The conference celebrating ‘The end of kuru: 50 years of research into an extraordinary disease’ was held in the Kohn Room of the Royal Society, London on 11–12 October 2007. It was an extraordinary meeting that is reflected in the proceedings published in this issue of the *Philosophical Transactions of the Royal Society*. There were 90 participants from around the world (figure 1).

The first day was devoted to reminiscences and reflections, personal and scientific, about kuru, about working and living in the kuru-affected region, and about undertaking research on kuru, from the perspectives of both medical scientists and the Fore people. On the second day, the results of contemporary research on kuru and their ever widening implications in neurodegeneration and beyond were presented, with discussions on bovine spongiform encephalopathy (BSE), its human counterpart variant Creutzfeldt–Jakob disease (vCJD) and other prion diseases.

The fundamental molecular processes involved in propagation of the transmissible agent or prion are now known to be of relevance in understanding the common degenerative brain diseases (such as Alzheimer's and Parkinson's diseases) and possibly normal brain ageing, and may explain a range of biological phenomena as evidenced by the emergence of the field of yeast and fungal prions. Indeed, the existence of multiple strains of prions implies non-Mendelian protein-based inheritance, with major evolutionary implications.

The proceedings represent the full diversity of the meeting and, unusually, include a good measure of history as well as science. This Introduction gives an account of the meeting; an account of kuru itself may be found in these proceedings in the introduction to the paper by Collinge *et al*. (2008).

All the scientists currently working on kuru attended the conference. The two Nobel laureates in the field, D. Carleton Gajdusek and Stanley Prusiner, both participated. There were 15 Papua New Guinean participants, 12 of whom were from the Fore linguistic group, the principal sufferers from kuru since the epidemic began about a century ago. Also attending were Peter Siba, the Director of the Papua New Guinea Institute of Medical Research, which has been involved in research on kuru since its inception in 1968, Adolf Saweri, the Chairman of the Council of the Institute, who as a young doctor had worked at Okapa, the government station at the centre of the kuru-affected region (Saweri 2008), and Ken Boone, a doctor in Goroka, capital of the Eastern Highlands Province, who in 2003 performed the last autopsy on a kuru patient (Boone 2008).

Although many of those who had worked on kuru in the field or laboratory were unable, owing to death or infirmity, to attend the conference, representatives of every era of kuru research participated. Systematic research on the disease began when Carleton Gajdusek joined Vincent Zigas in March 1957 (Gajdusek & Zigas 1957). They teamed up with Jack Baker, the patrol officer in charge of the government station at Okapa, and lived in his house (see fig. 1 in Reid 2008). Vin, sadly, has died and Jack was unable to attend the meeting, but Carleton was there in full force (Gajdusek 2008*a*,*b*). Other members of this team in 1957 were Lois Larkin Baker, who was unable to attend, and Lucy Hamilton Reid, who talked about her studies on the nutrition of the Fore (Reid 2008). Unfortunately, no members of the Adelaide group who had worked on kuru in the early years were able to be present, though we are fortunate to have the reminiscences of J. Henry Bennett and Donald Simpson in these proceedings (Bennett 2008; Simpson 2008). Cyril Curtain, who contributed to the first laboratory studies on kuru (Curtain 2008), took part in the meeting, which was very pleasing, though we missed the presence of Chev Kidson and the late Roy Simmons from this era of kuru research.

The research workers who followed next were Robert Glasse and Shirley Lindenbaum, who studied the anthropology of the Fore people and their neighbours, and Michael Alpers. Though Bob, sadly, has died, we were pleased that Shirley could attend and contribute to the proceedings of both the days (Lindenbaum 2008*a*,*b*). Of the doctors who worked at the Okapa Hospital at this time, Jonathan Hancock attended the meeting and Werner Stöcklin contributed to these proceedings (Stöcklin 2008).

The next group to undertake research in the field included Richard W. Hornabrook and Alex Nilsson, who were unable to attend; however, Dick was represented by Annette Beasley and we are pleased to have some reflections on kuru from him in the proceedings (Beasley 2008). John Mathews, Ray Spark and Coralie Mathews all participated in the meeting. Coralie gave her own reminiscences and reflections, which were, moreover, intended to be representative of the experiences of other wives who had not only supported their scientist husbands in the field but also made their own independent relationships with the Fore people that are still affectionately remembered in the area today (Mathews, C. 2008). Inamba Kivita, who had worked closely with Shirley Lindenbaum and John Mathews, was delighted to see his old friends again after so many years (Kivita 2008).

Richard Hornabrook became the first Director of the Papua New Guinea Institute of Medical Research and the Institute thereafter assumed the main responsibility for kuru epidemiological surveillance. John Cochrane, Margaret Cochrane, Donald Moir and Hilary King worked in the field during the late 1960s and early 1970s. Throughout the period from 1957 to 1985, Carleton Gajdusek made many return visits to the kuru-affected region and Michael Alpers, working from Perth in Australia, made annual field excursions from 1969 to 1976. They both contributed to kuru field surveillance and assisted all fieldworkers with updated printouts from the kuru database maintained at the National Institutes of Health in Bethesda by Judith Farquhar (who attended the meeting) and Steven Ono.

In 1977, Michael Alpers took over as Director of the Papua New Guinea Institute of Medical Research and the field surveillance intensified; Stanley Prusiner, Robert Klitzman and Phillip Tarr attended from these years (Prusiner 2008; Klitzman 2008; Tarr 2008). Phil Tarr conducted a village-based autopsy during his time in the field. Another autopsy was carried out by Euan Scrimgeour in Rabaul, since a Fore man came down with kuru after living there for many years and decided that he would die there (Scrimgeour 2008). The four most experienced field officers from that period, Auyana Winagaiya, the late Anua Senavaiyo, Igana Alesagu and Kabina Yaragi, were unable to attend but Anua's wife Andemba took part (Anua 2008). Finally, in 1996, field activities were enhanced by a collaboration between Michael Alpers and John Collinge, initially supported by the Wellcome Trust in London (of which John was then a Principal Research Fellow), and Jerome Whitfield was recruited to work in Papua New Guinea (Collinge 2008; Whitfield 2008). This collaboration then formed part of the newly formed MRC Prion Unit in London from 1998, directed by John Collinge. Dafydd Thomas and Edward McKintosh were involved in the fieldwork from the MRC Unit. Henry Pako outlined the current field activities of the kuru project (Pako 2008). Bridget Ogilvie, former Director of the Wellcome Trust, described the Trust's enthusiastic and flexible attitude to the project and their early support for John Collinge.

Of the neuropathologists who made the early significant observations on the histopathological features of kuru, Malcolm Fowler, E. Graeme Robertson and Elisabeth Beck had died and Igor Klatzo, who was invited to the meeting, died before it began. Their contributions were recognized by many participants during the course of the conference.

As a consequence of seeing the neuropathological features of kuru at an exhibition in the Wellcome Medical Museum in London in 1959, William J. Hadlow made the seminal connection between kuru and scrapie. Though he was unable to attend, we are pleased to have his reminiscences and reflections in these proceedings (Hadlow 2008). A carefully planned experiment to test the transmissibility of kuru to chimpanzees followed from Hadlow's observations within a few years and led to a successful outcome, which was reported in 1966 by Carleton Gajdusek, C. Joseph Gibbs and Michael Alpers (Gajdusek *et al*. 1966). Sadly, Joe Gibbs has died but his major contribution was honoured at the meeting. This work initiated our understanding of the human transmissible spongiform encephalopathies. Subsequent work by Stanley Prusiner and others led to the unifying concept of the prion diseases.

In October 2007, the epidemic of kuru may not have been entirely over but the end was certainly in sight. There was no patient with kuru in 2006 nor, as we now know, in 2007. The continuing field surveillance (Pako 2008) will tell us whether we have seen the last case; at the most we can expect only one or two more. This dramatic decline from 200 deaths a year over the first 5 years of kuru investigation is a cause for celebration. To be able to celebrate the disappearance of a fatal disease, especially one so distressing in its manifestations and so well documented, is an extraordinary experience and there was a sense of elation throughout the meeting. We were uniquely privileged at this celebration to have a good representation of the people who had suffered from the disease. The Fore participants had all lost close family members to kuru, including husbands, wives, children and mothers. Their suffering and resilience were expressed in their talks, which symbolized for the whole conference what all the people of the kuru-affected region over the span of a century (a population of approx. 40 000 at the peak of the epidemic) had endured (Bavasa 2008; Mabage 2008; Ombeya 2008; Poki 2008; Puwa 2008). The burden of kuru at its peak would be equivalent in the US, for example, to well over a million deaths per annum. Moreover, since the deaths were not evenly distributed in the total affected population and since most of the deaths were in adult women, in communities of high incidence the cumulative burden of mortality affected every family: in Fore society, nobody was untouched by kuru. Many of the scientific talks also paid tribute to the Fore people and their neighbours affected by kuru. A brief film by Rob Bygott and Ben Alpers, from a larger documentary in the making, showed Fore perspectives on kuru and gave a moving portrayal of both the suffering and the resilience exhibited by dying kuru patients and their many carers. To enhance the Fore participants' involvement in the meeting, at the end of each session the talks and discussion were briefly summarized, with typical Papua New Guinean oratory, in Tok Pisin (by Peter Siba or Ray Spark) and Fore (by Henry Pako or Anderson Puwa).

The meeting celebrated 50 years of scientific research on kuru and its many achievements. These achievements were reviewed within their historical context and their contemporary implications were analysed during the scientific presentations of the meeting. Social and behavioural studies of the Fore people, the interactions between the resident research workers and the local people, and the creative cross links that were established between disciplines during the field research were discussed in two papers, one on each day of the meeting, by Lindenbaum (2008*a*,*b*). Mathews, J. D. (2008) described how kuru had spread among the Fore, the trajectory of which has now been reasonably well established. The two factors that may have affected the probability of transmission are variations in mortuary practices (since the mode of transmission of the prion agent was through the consumption of dead relatives by the women and young children) and human genetic variation. Jerome Whitfield described recently acquired knowledge about Fore mortuary practices and their links with Fore cosmology (Whitfield *et al*. 2008). Much new work has been done on genetic variation and selection in relation to prion diseases, and the work on kuru, though it may not explain the local geographical spread of kuru, has produced results in population genetics that have been far-reaching and exciting (Mead *et al*. 2003, 2008).

Historical aspects of the epidemiology of kuru, including his own many significant contributions, were presented on the first day by Mathews, J. D. (2008). The sweep of the kuru epidemic from 1957 to the present, and the essential clues it has provided to solve the puzzle of kuru, were recounted on the second day by Michael Alpers from his personal experiences over the last 46 years (Alpers 2008*a*). John Collinge discussed the contemporary implications of kuru, in particular for vCJD, the human form of BSE (Collinge *et al*. 2008). When the recent detailed clinical and epidemiological findings on kuru, including evidence that incubation periods after oral transmission may be greater than 50 years, are combined with new genetic information a powerful dataset is created for exploring the wider implications of kuru for other human diseases (Collinge *et al*. 2008).

Broad vistas of science were opened up by Prusiner on prions, Gajdusek (2008*a*) on self-propagating proteins and other entities, Per Westermark on amyloidosis (Westermark & Westermark 2008) and Colin Masters on kuru and Alzheimer's amyloid plaques (paper not available for publication).

Though research on kuru has been ongoing for 50 years, only recently has it been possible to undertake molecular and biological strain typing of the prion agent of kuru. Studies at the MRC Unit have shown that kuru is caused by prion strains closely similar to those causing sporadic and iatrogenic CJD and quite distinct from that causing variant CJD (Wadsworth *et al*. 2008).

The neuropathology of the most recent autopsied case of kuru was presented in comprehensive detail by Sebastian Brandner (Brandner *et al*. 2008). We were fortunate to have other neuropathologists at the meeting, including Byron Kakulas and Catriona McLean. Byron's work was done during the second wave of the neuropathological study of kuru (Kakulas 2008) and Catriona's work was published during the last decade, from the examination of archival material held in Melbourne, which she has re-examined for these proceedings (McLean 2008). In 1967, Gabriele Zu Rhein produced the first electron micrographs of kuru. Though she was unable to attend the meeting, she has submitted a brief account of her early findings (Zu Rhein 2008).

We were privileged to have at the meeting not only Carleton Gajdusek in excellent form, despite recent ill-health, but also many of his associates ranging over a wide span of years of kuru research. The oldest were Taka Gomea, Tiu Pekiyeva, Tarubi Taguse and Koiye Tasa from the earliest years of research in the field, and their reunion with Carleton was an emotional experience shared by all who were fortunate to be witnesses (Gomea 2008; Pekiyeva 2008; Taguse 2008; Tasa 2008). We sorely missed the ebullience of Vin Zigas at the meeting but were delighted that his second wife (and widow) Jettie Zigas was able to attend and speak. Of Carleton's later associates Michael Alpers, David Asher, Richard Benfante, Judith Farquhar and Robert Klitzman participated (Alpers 2008*b*; Asher 2008; Benfante 2008; Farquhar 2008; Klitzman 2008) and Françoise Cathala, though unable to be present, submitted her reminiscences (Cathala 2008). Ceridwen Spark gave an animated account of Carleton's adopted family, with its strong Melanesian connections and links to kuru research.

Kuru has historical significance, not only for those who lived through the epidemic and experienced its horrors but also for historians of science (Anderson 2008), particularly those interested in human behaviour, in the transition from a traditional mode of life to the modern world, in the relationship of scientists to the people they are studying or whose diseases they are studying, and in the international politics of science (Scragg 2008).

Kuru has a scientific significance that has never been lost over the years, though its focus has changed, from the challenge of an exotic new disease reaching epidemic proportions in a restricted area of the tropics, to the first human transmissible spongiform encephalopathy, which led very quickly to the transmission of Creutzfeldt–Jakob disease (Gibbs *et al*. 1968), to a model for multidisciplinary epidemiological enquiry, to a model for intraspecies recycling, a lesson not learned that allowed the same augmenting mechanism to cause the cattle BSE epidemic, and to a model for the oral transmission of prion disease to humans (vCJD). The intracerebral transmission to chimpanzees had an incubation period of 2 years (Gajdusek *et al*. 1966), which halved on first chimpanzee-to-chimpanzee passage (Gajdusek *et al*. 1967): these were considered extraordinarily long incubations when they were first reported. Now we have recent work demonstrating incubation periods exceeding half a century after intraspecies oral transmission, with a strong dependence on host genetics. These findings in kuru will continue to have long-standing significance for neurology, infectious disease and public health. Kuru is indeed an extraordinary disease.

## Figures and Tables

**Figure 1:**
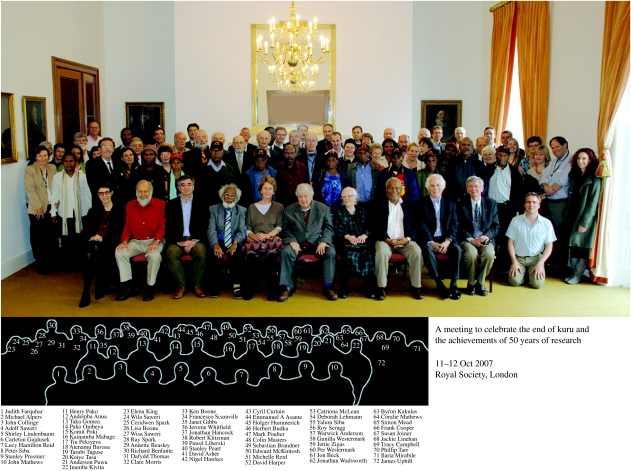
Group photograph of participants at the End of Kuru Conference.
